# Breaking the silence: Addressing sexual health challenges among migrant and refugee women

**DOI:** 10.1177/17455057251331263

**Published:** 2025-04-18

**Authors:** Zohra S Lassi, Negin Mirzaei Damabi, Mumtaz Begum, Jodie C Avery, Salima Meherali

**Affiliations:** 1 Robinson Research Institute, University of Adelaide, Adelaide, SA, Australia; 2 School of Public Health, Faculty of Health and Medical Sciences, University of Adelaide, Adelaide, SA, Australia; 3 Life Course and Intergenerational Health Research Group, Faculty of Health and Medical Science, University of Adelaide, Adelaide, SA, Australia; 4 Adelaide Medical School, Faculty of Health and Medical Sciences, University of Adelaide, Adelaide, SA, Australia; 5 Faculty of Nursing, University of Alberta, Edmonton, AB, Canada

**Keywords:** sexual function, sexual health, migrant, refugee

## Abstract

This editorial addresses the critical yet often overlooked issue of sexual health among migrant and refugee women. With nearly half of the world’s 281 million international migrants being women, their unique health challenges demand urgent attention. As a conceptual discussion, this editorial does not present empirical data but rather synthesizes existing literature and expert insights to explore the multifaceted barriers these women face, including financial constraints, language obstacles, cultural taboos, and social exclusion. We examine the complex interplay between acculturation and sexual function, emphasizing how cultural transitions influence sexual well-being. The discussion explores how cultural background shapes sexual attitudes, highlighting the need for culturally sensitive approaches in healthcare delivery. We propose multifaceted solutions, including developing culturally competent healthcare services, implementing targeted education programs, and improving research methodologies. This editorial aims to break the silence surrounding these issues and calls for concerted efforts to address the sexual health needs of migrant and refugee women, ultimately fostering healthier, more equitable societies.

## Plain language summary

Understanding and Supporting the Sexual Health Needs of Migrant and Refugee Women

Many women who move to new countries as migrants or refugees face unique challenges with their sexual health. Our editorial discusses these challenges and suggests ways to help.

In an era marked by unprecedented global migration, the world faces a pressing challenge that often remains shrouded in silence: the sexual health and well-being of migrant and refugee women. Of an estimated 281 million international migrants worldwide as of 2020, and over 35 million refugees at the end of 2022, nearly half of whom are women.^1^

Addressing the sexual health challenges of this population is essential for fostering equity in healthcare. This editorial is a conceptual discussion and does not present findings from empirical research. Instead, it synthesizes insights from the existing literature and the authors’ experiences to explore the barriers, cultural complexities, and potential solutions related to sexual health among migrant and refugee women.

Migrant and refugee women grapple with a complex web of challenges that impede their sexual health. Financial hardship limits their access to healthcare, while language barriers obstruct effective communication with healthcare providers,^2^ leading to mistrust and inadequate care. Social exclusion and discrimination based on ethnicity, race, religion, culture, and nationality further compound these issues,^3^ often rendering these women isolated and underserved. Moreover, existing efforts surrounding sexual and reproductive health often emphasize adverse outcomes, such as Sexually Transmitted Infection (STI) and Human Immunodeficiency Virus (HIV), unintended pregnancies, and sexual violence, reflecting deeply rooted cultural and political sensitivities.^4,5^ These sensitivities are further intensified by cultural and religious beliefs, with many societies considering discussion around sexual health taboo or forbidden. Such cultural reticence not only suppresses open dialog but also impedes the pursuit of essential care, education, and support.^6,7^ Compounding these issues is the trauma associated with displacement, which can have lasting effects on sexual health and overall mental and physical well-being.^8,9^

These multifaceted challenges unfold against a backdrop where sexual health issues are already prevalent in the general population. Studies indicate that around 40% of women worldwide report some form of sexual dysfunction.^10^ However, the specific experiences and prevalence of sexual dysfunction among migrant and refugee women remain largely understudied and underreported.^11^ This gap in knowledge underscores a critical oversight in both research and healthcare practice, where immediate survival needs often overshadow essential aspects of long-term health and quality of life, such as sexual well-being.^12^

Sexual satisfaction is a crucial aspect of overall well-being, yet its expression and experience are deeply influenced by cultural context.^13^ For migrant women, navigating between their cultural heritage and the norms of their new environments can create conflicting feelings and expectations around sexuality. Research on sexual pleasure among migrant women presents a complex picture with conflicting results.^14^ While some research indicates similar levels of satisfaction between migrant and native populations, others report lower satisfaction among migrants.^15^ These disparities can be attributed to various factors, including the stress of adapting to a new culture, differences in societal attitudes toward sex, and the preservation of traditional beliefs that may conflict with more liberal perspectives encountered in their host countries. The struggle to balance these divergent influences can lead to confusion, guilt, and decreased satisfaction, highlighting the need for culturally sensitive support systems that acknowledge and respect this delicate balancing act.^16^

A consistent trend emerges across studies examining sexual desire and arousal: migrant women frequently report lower levels compared to their native counterparts.^17^ This pattern raises critical questions about how the migration experience impacts sexual expression and enjoyment.^18^ Moreover, feelings of cultural dissonance and sexual guilt, often stemming from conservative upbringings, can inhibit the expression and enjoyment of sexuality in a new cultural context. These emotional and psychological burdens underscore the profound impact that the migration journey can have on intimate aspects of life, suggesting a pressing need for supportive interventions that address not only physical health but also emotional and psychological well-being.^19^

The process of acculturation—adapting to and adopting aspects of a new culture—plays a significant role in shaping the sexual experiences of migrant women.^20^ The relationship between acculturation and sexual function is complex and multifaceted. For some, greater integration into the mainstream culture correlates with increased sexual desire and arousal, possibly due to exposure to more open attitudes toward sexuality and better access to sexual health resources.^21^ Conversely, others may experience little to no change, or even negative impacts, as they struggle with maintaining their cultural identity while adapting to new norms. This variability highlights that acculturation is not a uniform process and underscores the importance of personalized approaches in sexual healthcare that consider individual experiences and levels of cultural integration.^22^ This variability underscores the complex nature of cultural adaptation and its effects on sexuality, highlighting the need for nuanced approaches to sexual health care for migrant women.

Cultural background significantly influences sexual attitudes and knowledge. Many migrant women come from societies where sex education is limited or non-existent, leading to conservative attitudes and a lack of understanding about sexual health and rights.^23^ This knowledge gap can result in misconceptions, unsafe practices, and reluctance to seek care or discuss sexual issues openly.^24^ Addressing this requires comprehensive and culturally appropriate sexual health education that respects and acknowledges diverse cultural values while providing accurate and accessible information. Healthcare providers and educators must work collaboratively to create inclusive programs that empower migrant women with the knowledge and confidence to take charge of their sexual health.

Confronting the sexual health challenges faced by migrant and refugee women demands a concerted and multifaceted effort. Developing culturally competent healthcare services is paramount, ensuring that providers are trained to understand and respect the diverse backgrounds and experiences of their patients.^25^ Equally important are creating social support systems and enacting supportive policies for migrant and refugee women. Targeted education programs must be implemented to provide accessible and relevant information, fostering environments where open and respectful dialog about sexual health is possible. Improving research methodologies to gather more accurate and representative data will inform better policymaking and resource allocation. Additionally, establishing robust social support systems and enacting policies that protect and promote the health rights of migrant women are critical steps toward meaningful change.

The journey toward addressing and improving the sexual health of migrant and refugee women is undoubtedly challenging but essential. Breaking the silence surrounding these issues is the first step toward fostering healthier, more equitable societies. It requires acknowledging the complex interplay of cultural, social, and psychological factors that influence sexual health and committing to actions that address these dimensions comprehensively. Are we ready to confront these challenges head-on? Our collective response will not only shape the health outcomes of millions of women but will also reflect our commitment to inclusivity, equity, and human dignity. By embracing this responsibility, we can pave the way for a future where all individuals, regardless of their migration status, have the opportunity to achieve optimal sexual health and well-being.

## Statements and declarations

**Ethics approval and consent to participate:** Ethics approval was not required for this editorial as it does not involve human participants or animal subjects.

**Consent for publication:** Not applicable as this editorial does not contain data from any individual person.
